# Type III Secretion-Dependent Sensitivity of Escherichia coli O157 to Specific Ketolides

**DOI:** 10.1128/AAC.02085-15

**Published:** 2015-12-31

**Authors:** Romina J. Fernandez-Brando, Nao Yamaguchi, Amin Tahoun, Sean P. McAteer, Trudi Gillespie, Dai Wang, Sally A. Argyle, Marina S. Palermo, David L. Gally

**Affiliations:** a Division of Immunity and Infection, The Roslin Institute and R(D)SVS, The University of Edinburgh, Easter Bush, Midlothian, United Kingdom; b Laboratory of Pathogenesis and Immunology of Infectious Diseases, IMEX-CONICET, National Academy of Medicine, Buenos Aires, Argentina; c Faculty of Veterinary Medicine, Kafrelsheikh University, Kafrel-Sheikh, Egypt; d IMPACT Facility, Centre for Integrative Physiology, The University of Edinburgh, Edinburgh, United Kingdom; e State Key Laboratory of Molecular Vaccinology and Molecular Diagnostics, Xiamen University, Xiamen, Fujian, People's Republic of China

## Abstract

A subset of Gram-negative bacterial pathogens uses a type III secretion system (T3SS) to open up a conduit into eukaryotic cells in order to inject effector proteins. These modulate pathways to enhance bacterial colonization. In this study, we screened established bioactive compounds for any that could repress T3SS expression in enterohemorrhagic Escherichia coli (EHEC) O157. The ketolides telithromycin and, subsequently, solithromycin both demonstrated repressive effects on expression of the bacterial T3SS at sub-MICs, leading to significant reductions in bacterial binding and actin-rich pedestal formation on epithelial cells. Preincubation of epithelial cells with solithromycin resulted in significantly less attachment of E. coli O157. Moreover, bacteria expressing the T3SS were more susceptible to solithromycin, and there was significant preferential killing of E. coli O157 bacteria when they were added to epithelial cells that had been preexposed to the ketolide. This killing was dependent on expression of the T3SS. Taken together, this research indicates that the ketolide that has accumulated in epithelial cells may traffic back into the bacteria via the T3SS. Considering that neither ketolide induces the SOS response, nontoxic members of this class of antibiotics, such as solithromycin, should be considered for future testing and trials evaluating their use for treatment of EHEC infections. These antibiotics may also have broader significance for treating infections caused by other pathogenic bacteria, including intracellular bacteria, that express a T3SS.

## INTRODUCTION

Type III secretion systems (T3SSs) are expressed by a cross-section of Gram-negative bacterial pathogens to export effector proteins out of the bacterium and often directly into host eukaryotic cells. These secreted effectors manipulate host cell processes presumably to the advantage of bacterial colonization and subsequent transmission. For enteropathogenic Escherichia coli (EPEC) and enterohemorrhagic E. coli (EHEC), the T3SS injects proteins into epithelial cells, thus reorganizing the actin cytoskeleton and allowing tight intimate binding to the cell surface, with the subsequent formation of typical attaching-and-effacing (A/E) lesions. A cocktail of other effector proteins then controls host cell innate responses to prolong this interaction (1, 2). The locus of enterocyte effacement (LEE) pathogenicity island encodes the EHEC T3SS and a subset of secreted effector proteins, while the remainder are encoded by prophage regions integrated at multiple sites around the genome (3). The LEE genes are encoded in 5 main operons (*LEE1* to *LEE5*), and their induction is controlled by a complex network of regulators that includes the LEE-encoded regulator (Ler), which is encoded at the start of the *LEE1* operon (4–7). The *LEE1*, *LEE2*, and *LEE3* operons encode components that span the inner and outer membranes, which include EscC, the outer membrane porin, and EscN, the ATPase of the system. The *LEE4* operon includes EspA and EscF, which form the filament and the needle structures, respectively (8); EspB and EspD, which form a pore in the host cell membrane (9); and, potentially, EspF, which is injected into the host cell and targeted to the mitochondria, where it participates in the cell death pathway (10). In addition, EspF has also been demonstrated to disrupt transepithelial cell resistance, leading to disruption of tight junctions (11). Tir and intimin are the proteins that determine intimate attachment to the host epithelium and are encoded on the *LEE5* operon, together with CesT, a chaperone for Tir (4, 12, 13).

For pathogens expressing T3SSs, these are generally essential for virulence and have been the focus of specific antivirulence or pacification compounds that can limit the expression or activity of the T3SS (14, 15). These compounds have been shown to be broadly effective against a number of pathogenic bacteria that utilize T3SS, such as EHEC (16), EPEC (17), Salmonella enterica serovar Typhimurium (18), Chlamydia spp. (19), and Yersinia pseudotuberculosis (20).

In the case of EHEC infection, there is a concern that any antibiotic treatment could induce the production of Shiga toxin (Stx), the main factor associated with kidney damage and the life-threatening consequences of human EHEC infections. The genes for Stx are encoded within the late-gene region of temperate bacteriophages integrated in the bacterial chromosome (21, 22). The phage late genes encode proteins responsible for viral replication, assembly, and lysis of the host E. coli cell. These genes are silent during lysogeny and become expressed only during the lytic cycle. Both Stx and new viral particles are released when the bacteria undergo lysis. The switch from lysogeny to the lytic cycle is controlled by the bacterial SOS stress response (23), which is induced by certain antibiotics (24–27). As Stx variants are the key pathogenic factors that lead to life-threatening systemic complications in people infected with EHEC strains, Stx phage induction by any antibiotic treatment should be investigated. Although certain classes of antibiotics are known to induce SOS responses, other antibiotics have successfully been used in outbreaks (28).

The effects of different classes of antibiotics at sub-MICs have received various levels of attention (29), but it is important to know if certain antibiotics can have added functionality by repressing virulence at concentrations that would not normally prevent bacterial growth.

In this study, we initially screened for bioactive compounds that have an effect on expression of the EHEC T3SS but a limited impact on bacterial growth. This screening identified the ketolide telithromycin. Subsequent research on a derivative known to be less toxic in humans, solithromycin, demonstrated not only that both ketolides inhibit translation of the T3SS at concentrations that still allow bacterial growth but also that bacteria expressing a T3SS are more sensitive to solithromycin. E. coli O157 isolates expressing a T3SS were recovered at significantly lower levels than a T3SS mutant when they were added to epithelial cells that had been preexposed to solithromycin, indicating that the antibiotic may be entering the bacteria through the secretion system during the infection process.

## MATERIALS AND METHODS

### Bacterial strains and media.

The bacterial strains used in this study are described in Table 1. Bacteria were cultured in Luria-Bertani (LB) broth or minimal essential medium (MEM)-HEPES (Sigma-Aldrich) supplemented with 0.1% glucose and 250 nM Fe(NO_3_)_3_. Caco-2 cells were grown in Dulbecco modified Eagle medium (DMEM) supplemented with 10% fetal bovine serum (FBS), 15 mM l-glutamine, and 1% penicillin-streptomycin (Sigma-Aldrich). When measuring the promoter activity of *sulA*::*gfp*, strains were cultured in M9 medium (Sigma-Aldrich) supplemented with 0.2% glucose, 2 mM MgSO_4_, and 0.1% acid-hydrolyzed Casamino Acids. When required, antibiotics were added to the media at the following final concentrations: 1 μg/ml for mitomycin C (MMC), 0.03 and 0.10 μM for chloramphenicol, 1.10, 1.48, and 1.85 μM for telithromycin; and 0.25, 0.50, 0.75, 1.00, 3.00, and 5.00 μM for solithromycin (Cempra Pharmaceuticals). In order to assess the impact of solithromycin on the viability of bacteria expressing or not expressing a T3SS, ZAP193 and its Δ*LEE2* derivative (Table 1) were cultured overnight in LB and then inoculated into MEM-HEPES to an optical density at 600 nm (OD_600_) of 0.5, at which point 3 μM solithromycin was added to the cultures and their optical densities were measured at 30-min intervals for 150 min.

**TABLE 1:** Bacterial strains and plasmids used in this study

Strain or plasmid	Description	Source or reference
Strains		
ZAP193	Stx negative, NCTC 12900	33
ZAP198	EHEC O157:H7 strain Walla 3 Nal^r^	74
Δ*LEE2*	ZAP193 derivative with *LEE2* deletion	This study
ZAP1004	ZAP198 derivative with *ler* deletion	75
TUV93-0	EHEC O157:H7 strain EDL933 *stx* negative derivative	32
Plasmids		
pKC26	Promoter-less GFP plasmid	76
pKC26*sulA*	GFP fusion plasmid with promoter from *sulA*	This study
pDWLEE1	GFP fusion plasmid with promoter from *LEE1* operon	This study
pDWLEE2	GFP fusion plasmid with promoter from *LEE2* operon	This study
pDWLEE3	GFP fusion plasmid with promoter from *LEE3* operon	This study
pDW6	GFP fusion plasmid with promoter from *LEE4* operon	79
pDWLEE5	GFP fusion plasmid with promoter from *LEE5* operon	This study
pAJR145	pACYC *rpsM*::*gfp*	34
pAJR71	pACYC *LEE1*::*gfp*	33
pWSK29	Low-copy-no. vector, Amp^r^	77

### NINDS library.

The National Institute of Neurological Disorders and Stroke (NINDS) library is a collection of 1,040 known bioactive compounds. It is a small-scale library that may be feasibly screened in general laboratory facilities. It comprises a wide range of drugs that are used clinically. Many of the compounds are FDA-approved drugs, and their properties are well established (30, 31). They have all passed major safety and clinical trials (30). The library was supplied to us by the Centre for Therapeutics Discovery (CTD) at the Medical Research Council (MRC). See http://www.mrctechnology.org/about/our-structure/centre-for-therapeutics-discovery for further details. The compounds in the NINDS library cover a wide range of therapeutic effects. Out of the 1,040 compounds, there are 157 antibacterials, which account for the largest single group of compounds in the library. This is followed by anti-inflammatories and antineoplastics, with 83 and 76 compounds, respectively. The rest of the library comprises many smaller groups with different therapeutic effects (e.g., antidepressants, muscle relaxants, antihistamines, diuretics, sweeteners, sunscreens). The stock plates of the NINDS library are prepared at a single concentration of 1 mM, and the molecular weights of the compounds in the library vary widely. Screening was at a single concentration recommended by the suppliers, 10 μM in 1% dimethyl sulfoxide. The NINDS library was screened with E. coli O157:H7 TUV 93-0, a Stx phage-negative version of E. coli O157 EDL933 that has a relatively high level of T3SS expression (32). This strain was transformed with either pAJR71 (an LEE1 translational reporter [33]) or pAJR145 (an RpsM reporter [34]). The library was screened twice with each assay. The screening conditions were defined from a number of preliminary experiments that examined different plate formats, culture volumes, and types of aeration. This NINDS library was screened in a 96-well plate format with each compound predispensed into each well (10 μl). The bacterial cultures (transformed with either the LEE or RpsM readout) in M9 glucose medium (100 μl) were dispensed into the wells at an OD_600_ of 0.2 using a Thermo Scientific multidrop dispenser. The plates were then secured in moist boxes and incubated with rotation at 37°C for 6 h. The fluorescence readings were taken on a FLUOstar Optima reader (BMG Lab Tech). Assay assessment was based on conventional criteria applied to high-throughput sequencing (HTS) screens. High *Z*′ values were obtained at an endpoint of 6 h for the RpsM reporter (*Z*′ > 0.80), although more variability was shown for the LEE1 reporter (*Z*′ = 0.40).

### Preparation of T3S culture supernatant proteins and analysis by SDS-PAGE.

Bacteria were grown overnight in LB medium, diluted in MEM-HEPES supplemented with glucose and iron, and then incubated at 37°C until an OD_600_ of ∼0.9 was reached. The cultures were centrifuged at 4,000 × *g* for 30 min, and the supernatants were passed through a 0.45-μm-pore-size low-protein-binding filter (Millipore). Proteins were precipitated by the addition of 10% (vol/vol) trichloroacetic acid (TCA; Sigma-Aldrich) and bovine serum albumin (BSA; 4 μg/ml; NEB) at 4°C overnight. Solutions were centrifuged at 4,000 × *g* for 30 min at 4°C, the supernatants were carefully poured off, and the protein pellets were air dried. The pellets were resuspended by the addition of an appropriate volume of 1.5 M Tris-HCl, pH 8.8. Culture supernatant proteins were subsequently separated through a 12% SDS-polyacrylamide gel and imaged with colloidal blue staining (Severn Biotech). Gel images were captured using a Flowgen MultiImage light cabinet and ChemiImager 4000i (v.4.04) software.

### Western blotting.

To detect EspD by Western blotting, culture supernatant proteins were obtained as described above. For RecA detection, 1 ml of the same culture was centrifuged, the supernatant was discarded, and the cell pellet was suspended in 0.1 ml 2× Laemmli buffer (35). Appropriate volumes were incubated at 100°C for 5 min and then separated through a 10% SDS-polyacrylamide gel. Proteins were transferred onto a Hybond ECL nitrocellulose membrane (Sigma-Aldrich) using a Trans-Blot electrophoretic transfer cell (Bio-Rad), and membranes were blocked with 5% (wt/vol) milk powder (Sigma) in phosphate-buffered saline (PBS; Oxoid) at 4°C overnight. The membranes were sequentially incubated with 1:2,000 mouse monoclonal anti-EspD (a gift from Trinad Chakraborty, University of Giessen) or 1:10,000 anti-RecA (Enzo Life Sciences) and then 1:3,000 rabbit polyclonal anti-mouse IgG-horseradish peroxidase-conjugated antibodies (NEB), all of which were diluted in PBS containing 5% (wt/vol) milk powder. All incubations were carried out at room temperature for at least 1 h on a platform shaker and were washed for 3 times for 5 min each time in PBS before and after each antibody step. The membranes were visualized with chemiluminescent detection (GE Healthcare Life Sciences) on Hyperfilm ECL film (GE Healthcare Life Sciences) developed in a Protec automatic film processor (Optimax). Images were taken as described above.

### Cell binding assays.

Caco-2 cells were cultured for 14 days at 37°C in 5% CO_2_ and moisture. This amount of time allows the differentiation and development of microvilli. Caco-2 cells were seeded into 12-well plates at 10^5^ cells per well and incubated overnight. Caco-2 cell cultures were washed twice with MEM-HEPES without antibiotic 1 h before bacteria were added. Bacteria were inoculated from LB broth overnight cultures into prewarmed MEM-HEPES with or without the relevant antibiotic to an OD_600_ of ∼0.3, the bacterial cultures were diluted 1:20 into the corresponding medium, and 100 μl of bacterial suspension was added to each well of Caco-2 cultures (multiplicity of infection [MOI] = 20). The cells were incubated at 37°C in 5% CO_2_ in a moist box for 3 h. After this incubation period, the supernatants, which contained nonadherent bacteria, were discarded, the cells were washed three times with sterile PBS, and 100 μl of 0.1% Triton X-100 was added. The cells on each well were scraped off, and suspensions were serially diluted in PBS and triplicate plated onto LB plates. The plates were incubated overnight at 37°C, and colonies were counted the next day. For microscopy analysis, the bacteria were incubated for 1 h and the cells were washed twice with MEM-HEPES and incubated for 3 or 4 more hours. After this time, the cells were fixed using 4% paraformaldehyde (PFA) and permeabilized with 2% PFA–0.25% Triton X-100 at room temperature for 20 min. After three washes with PBS, samples were incubated overnight with rabbit anti-lipopolysaccharide (anti-LPS)–O157 antibody (MAST Group, Ltd.) at 1:4,000. After three more washes, samples were incubated with phycoerythrin (PE)-conjugated goat anti-rabbit immunoglobulin antibody at 1:1,000 (Molecular Probes) for an hour. Staining of F actin was carried out with fluorescein isothiocyanate (FITC)-phalloidin at 1:40 (Molecular Probes) for 90 min at room temperature on a platform shaker. The nuclei were stained with DAPI (4′,6-diamidino-2-phenylindole; Merck) for 10 min. The slides were washed three times with PBS and mounted with Hydromount (National Diagnostic), and coverslips were applied. The mounted slides were examined by confocal and fluorescence microscopy.

To determine the effect of pretreating epithelial cells on the binding and viability of E. coli O157, embryonic bovine lung (EBL) cells were used (http://www.dsmz.de). Confluent EBL cells were treated with 1 ml of MEM-HEPES with iron and glucose containing 5 μM (final concentration) solithromycin for 3 h. The cells were washed 3 times with medium without solithromycin and kept with this medium for 20 min before adding the bacteria. For this, fresh colonies of strain ZAP198 were inoculated into LB broth and incubated at 37°C and 200 rpm for 16 h. Saturated cultures were subcultured 1:100 in MEM-HEPES with iron and glucose and cultured until the OD_600_ was 0.3. One hundred microliters of the bacteria was added to each well (MOI, ∼100). The bacteria were incubated with the EBL cells for 3 h at 37°C in 5% CO_2_ at 80% humidity. At the required time, the cells were washed 6 times with PBS and treated with 0.2% (vol/vol) Triton X-100 (100 μl in each well; Sigma). The cells were removed by scraping, and serially diluted cell suspensions were plated onto LB agar plates. Following incubation at 37°C for 16 h, the bacterial colonies were enumerated. The bacteria were also counted by plating at the time of challenge to detect the initial numbers added to the cells. For competition of the T3S-positive or -negative strains, the Δ*ler* derivative of ZAP198 (ZAP1004) was transformed with pWSK29 to mark the strain with ampicillin resistance (Amp^r^). One hundred microliters each of this and the parent strain was added to the EBL cells, and the same method described above was followed. The strains were enumerated independently, as the wild-type strain is resistant to nalidixic acid and the *ler* deletion mutant is resistant to ampicillin.

### Live/dead staining.

EBL cells (3 × 10^5^) were seeded overnight on glass coverslips in 24-well plates precoated with murine collagen (Sigma) according to the manufacturer's instructions. On the next day, the cells were incubated with 1 ml of MEM-HEPES with iron and glucose with and without 5 μM solithromycin for 3 h. The cells were then washed 3 times with antibiotic-free medium. Bacteria were prepared as described above for the work on binding to EBL cells but were added at an MOI of 20. The infected cells were incubated for 3 h at 37°C in 5% CO_2_ at 80% humidity. The cells were washed twice with 3-(*N*-morpholino)propane sulfonic acid (MOPS)-MgCl_2_, and 0.5 ml LIVE/DEAD BacLight bacterial viability kit staining solution (Molecular Probes) was added to each well. LIVE/DEAD staining solution is 5 μM SYTO9 and 30 μM propidium iodide (final concentrations) in MOPS-MgCl_2_. Cells were incubated for 15 min at room temperature in the dark and washed three times in MOPS-MgCl_2_. Coverslips were inverted, placed face down onto glass slides, and sealed with clear nail polish. The images were acquired within 30 min, using a fluorescence microscope as detailed below.

### Measurement of LEE and SulA promoter activity.

In order to assess the expression of individual LEE operons, a series of plasmid-based promoter::green fluorescent protein (GFP) reporter fusions was constructed (Tables 1 and 2). The first genes, including their native promoters from each LEE operon, were amplified by high-fidelity PCR using the primer pairs listed in Table 2. The resulting PCR products were then digested and inserted into pAJR70 (Table 1) to create *LEE1* to *LEE5* translational fusions. All the final constructs were confirmed to be correct by sequencing. A plasmid constitutively expressing GFP (pAJR145 *rpsM*::*gfp*) was used as a control. These plasmids were transformed into ZAP198, and transformants were selected on LB agar containing 50 μg/ml chloramphenicol. Strains harboring these reporters were cultured with or without the antibiotic being assessed in MEM-HEPES supplemented with chloramphenicol, glucose, and iron, as indicated above, and the fluorescence produced by each bacterial population was measured every hour by transferring 100-μl aliquots of culture into triplicate wells in a black 96-well plate (Fluoro-Nunc) and reading the plate in a fluorimeter (FLUOstar Optima). The OD_600_ of the cultures was used to monitor growth. Fluorescence was plotted against the optical density by using GraphPad Prism (v.5.01) software. The promoter-less plasmid pKC26 (Table 1) was used to correct for the background fluorescence of the strain and medium.

**TABLE 2:** Primers used in this study

Primer	Plasmid	Sequence^*a*^
Lerpro5BamH	pDWLEE1-GFP	CGGGATCC GTTTATGCAATGAGATCTATC
Ler3kpn	pDWLEE1-GFP	GGGGTACCAATATTTTTCAGCGGTATTATTTC
sepZpro5BamH	pDWLEE2-GFP	CGGGATCCGCGTTTTCGTTATACTCTAAAGC
sepZ3kpn	pDWLEE2-GFP	GGGGTACCGGCATATTTCATCGCTAATGC
lee3pro5BamH	pDWLEE3-GFP	CGGGATCCAGAGCCGTAGTGGTAAGTGC
lee3kpn3	pDWLEE3-GFP	GGGGTACCTGATGTCATCCTGCGAACGA
Tirpro5Bgl	pDWLEE5-GFP	GAAGATCTGCTTCCTGGTGTATAGCATGG
Tir3kpn	pDWLEE5-GFP	GGGGTACCGACGAAACGATGGGATCCC
5′-PsulA-XbaI	pKC26*sulA*	AAAATCTAGAGGTATTCAATTGTGCCCAACG
3′-PsulA-XbaI	pKC26*sulA*	AAAATCTAGAAATCAATCCAGCCCCTGTG

a Restriction enzyme recognition sites are underlined.

To determine whether there was induction of *sulA* gene expression by the tested antibiotics, the *sulA* promoter (P*sulA*) was amplified using the primers 5′-PsulA-XbaI and 3′-PsulA-XbaI (Table 2). Restriction enzyme sites were introduced into the primers. The promoter region was initially cloned into the intermediate pJET1.2 plasmid (Fermentas), following the manufacturer's instructions. P*sulA* was subcloned into the unique XbaI site upstream of a GFP-positive gene in pKC26, thus constructing a transcriptional GFP reporter plasmid (Table 1). Using the primers specific for the region flanking the XbaI site and the primers specific for the promoter regions, the orientation of P*sulA* in the final pKC26 constructs was confirmed by PCR. This plasmid was then transformed into ZAP198, and ZAP198 *sulA*::*gfp* transformants were selected on LB agar containing 12.5 μg/ml chloramphenicol. Strains harboring this reporter were cultured in M9 medium supplemented with chloramphenicol, and the fluorescence produced by each bacterial population was measured 6 h later, as described above. Promoter-less pKC26 in the ZAP198 background acted as a control for autofluorescence. As a positive control, mitomycin C was added (1 μg/ml), given that it induces DNA damage and SulA expression.

### Microscopy.

Confocal data for A/E lesion analyses were acquired using a 1,024- by 1,024-pixel image size, a Zeiss Plan Apochromat 63× oil immersion lens (numerical aperture, 1.4), and a multitrack (sequential scan) experimental setup on a Zeiss LSM510 microscope. The live/dead imaging was carried out using a Leica DM LB2 microscope and a 40× objective lens. Images were captured using a Hamamatsu ORCA-ER black-and-white charge-coupled-device digital camera.

### Statistical analysis.

Data are expressed as the mean ± standard error of mean (SEM) or standard deviation (SD) and were analyzed for statistically significant differences by either a two-tailed Student's *t* test or analysis of variance (ANOVA) according to the number of experimental groups. ANOVA analyses were followed by a comparison between treatments performed by the Newman-Keuls multiple-comparison test.

## RESULTS

### Screening of the NINDS library for compounds that repress LEE1 expression.

The study analyzed the expression of an Ler (LEE1) translational fusion to GFP (pAJR71) in E. coli O157:H7 strain TUV-93 (Stx negative) in a 96-well plate format. This readout was compared to the expression of a transcriptional reporter to RpsM (pAJR145), a ribosomal protein that provides an indication of growth status (16). Preliminary studies (data not shown) indicated that a 6-h time point produced statistically robust measurements. Fluorescence expression from pAJR71 had a lower *Z*′ score (0.4) than that from pAJR145 (0.84) as a result of a relatively high standard deviation across the samples. Due to the relatively low *Z*′ value for the LEE1 screen, hits were defined as compounds which affected the fluorescence signal by 3 SDs or more. Under this definition, the pAJR145 (RpsM) screen had a hit rate of 36% and the pAJR71 (LEE1) screen had a hit rate of 16%.

Only three compounds, sulfamethoxazole, cefaclor, and telithromycin, showed more significant impacts (reductions) on the LEE1 signal than the RpsM signal. Telithromycin showed the greatest differential with a 5× SD reduction for the LEE1 reporter and a 1× SD reduction for the RpsM reporter (indicated by values of −5.0 and −1.0, respectively). The values for sulfamethoxazole were −3.1 and −1.7, respectively, while those for cefaclor were −3.5 and −1.3, respectively. Other macrolide/ketolide antibiotics in the NINDS library did not show the same level of specific LEE1 repression, with the SD values for the LEE1 and RpsM reporters being as follows: −6.7 and −23.2, respectively, for azithromycin; 1.7 and −2.7, respectively, for clarithromycin; 4.2 and −1.6, respectively, for dirithromycin; 2.0 and −4.6, respectively, for roxithromycin; 1.5 and −4.8, respectively, for erythromycin; −0.5 and −1.2, respectively, for oleandomycin; and 0.5 and −3.5, respectively, for spiramycin. It is important to point out that all of these antibiotics were tested only at a concentration of 10 μM on the initial screening. This final concentration was based on dilution of the stock plates supplied to us at a stated concentration of 1 mM.

### Analysis of type III secretion profiles.

**FIG 1:**
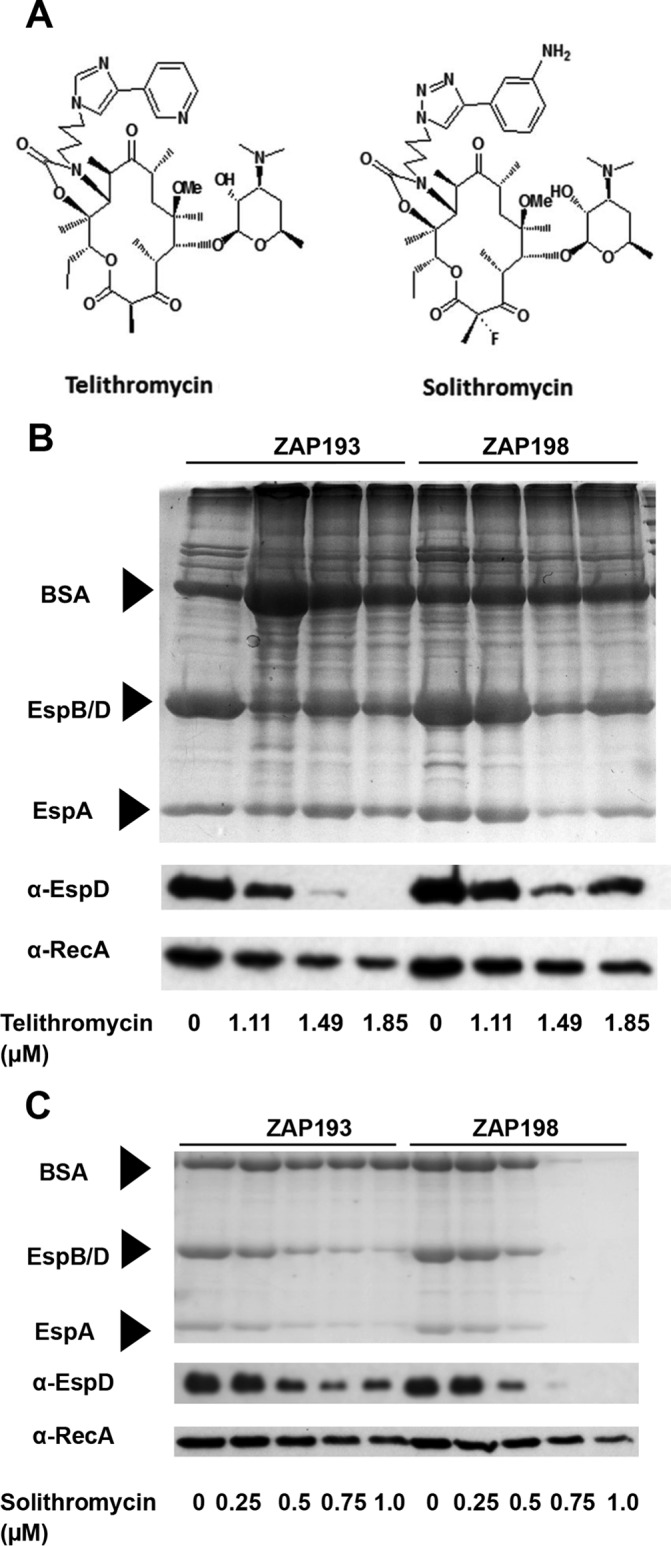
Telithromycin and solithromycin structures and effect on type III secretion. (A) Molecular structures of telithromycin and solithromycin (78). (B and C) Impact of increasing concentrations of ketolides on protein secretion by E. coli O157 strains ZAP193 and ZAP198 (Table 1) cultured in MEM-HEPES in the presence of the indicated concentrations of telithromycin (B) and solithromycin (C). Gels were stained with colloidal blue or transferred onto nitrocellulose membranes, and the EspD and RecA proteins were detected with specific antibodies, as indicated on the left. Arrows, bands corresponding to BSA (66.4 kDa; added to help precipitation), EspB/D (32.6 and 39.1 kDa, respectively), and EspA (20.6 kDa). Samples were prepared from equal volumes of bacteria (50 ml).

Telithromycin is associated with toxicity issues in humans, and there has been the development of new ketolides known as a fluoroketolides, of which solithromycin (Cempra Pharmaceuticals) is currently in clinical trials (36). Solithromycin is associated with greater potency and lower toxicity than telithromycin. It was therefore decided to investigate both telithromycin and solithromycin in terms of their capacity to inhibit T3S on the basis of the initial findings for telithromycin in the HTS. The structures of telithromycin and solithromycin are illustrated in Fig. 1A. On the basis of the results of the primary library screen, the impact of both telithromycin and solithromycin on T3SS protein secretion was determined for two O157:H7 Stx-negative E. coli strains (ZAP193 and ZAP198) at several sub-MICs of the antibiotics. Total secreted proteins were examined by colloidal blue staining followed by Western blotting for the secreted translocon protein EspD (Fig. 1B and C), as described in Materials and Methods. Both telithromycin (Fig. 1B) and solithromycin (Fig. 1C) reduced the overall level of secretion of proteins, including EspD, when used at concentrations ranging from 1.49 to 1.85 μM and from 0.5 to 1.0 μM, respectively. At the higher concentrations of these ranges, the antibiotics did have a slight impact on bacterial growth, as they extended the time taken to reach particular optical densities (Table 3).

**TABLE 3:** Antibiotic effect on bacterial growth

Bacterial strain	Telithromycin			Solithromycin		
	Concn (μM)	Time of sampling (h)	OD_600_^*a*^	Concn (μM)	Time of sampling (h)	OD_600_
ZAP193	0.00	4.25	0.83	0.00	5.00	0.72
	1.11	4.75	0.84	0.25	5.00	0.75
	1.48	4.75	0.76	0.50	5.50	0.70
	1.85	4.75	0.74	0.75	6.50	0.72
				1.00	6.50	0.59
ZAP198	0.00	4.25	0.88	0.00	5.00	0.78
	1.11	4.25	0.84	0.25	5.00	0.78
	1.48	4.25	0.80	0.50	5.50	0.81
	1.85	4.25	0.78	0.75	6.50	0.70
				1.00	6.50	0.57

a OD_600_, optical density at 600 nm.

### Reporter gene assays.

**FIG 2:**
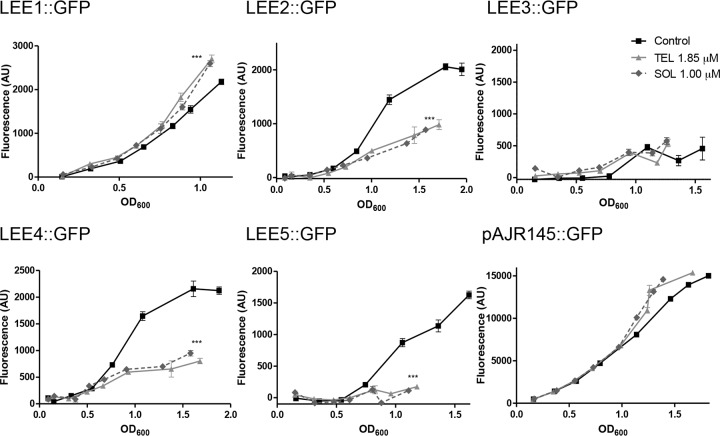
Analysis of LEE gene expression in the presence of ketolides by using GFP fusions. Individual promoters of each LEE operon were fused with GFP and transformed into E. coli O157:H7 strain ZAP198 (Table 1). Plasmid pAJR145 is a control fusion in which the *rpsM* promoter was fused with *gfp* (*rpsM*::*gfp*) and transformed into ZAP198. The fluorescence and OD_600_ were determined every hour. ZAP198 transformed with a promoter-less GFP plasmid (pKC26) was used as a background control. Lines represent the means of three biological repeats ± 1 SD. The results of one representative experiment out of at least three that were performed with similar results are shown. *P* < 0.005, ANOVA; ***, *P* < 0.001, Newman-Keuls multiple-comparison test, compared to the results for the control. OD_600_, optical density at 600 nm; AU, arbitrary units; TEL, telithromycin; SOL, solithromycin.

To test what effects both telithromycin and solithromycin have on expression of the five main polycistronic operons present on the locus of enterocyte effacement (LEE) that encodes the bacterial T3SS, published GFP translational reporter fusions to each operon promoter were tested in the presence of telithromycin at 1.85 μM and solithromycin at 1 μM. The *rpsM*::*gfp* (pAJR145) fusion was again used as a control, as it encodes a non-LEE-related ribosomal protein. While there was no evidence for repression of the LEE1 or RpsM reporter at these specific concentrations, both telithromycin and solithromycin significantly repressed LEE2, LEE4, and LEE5 expression (Fig. 2). LEE3 expression was difficult to assess, as it showed very low levels of expression in either the presence or absence of the antibiotics. These results indicate that the two ketolides have a preferential impact on expression of the proteins encoded by LEE operons *LEE2*, *LEE4*, and *LEE5* compared to that on the protein encoded by *LEE1* at these concentrations.

### Analysis of ketolides on adherence and A/E lesion formation.

**FIG 3:**
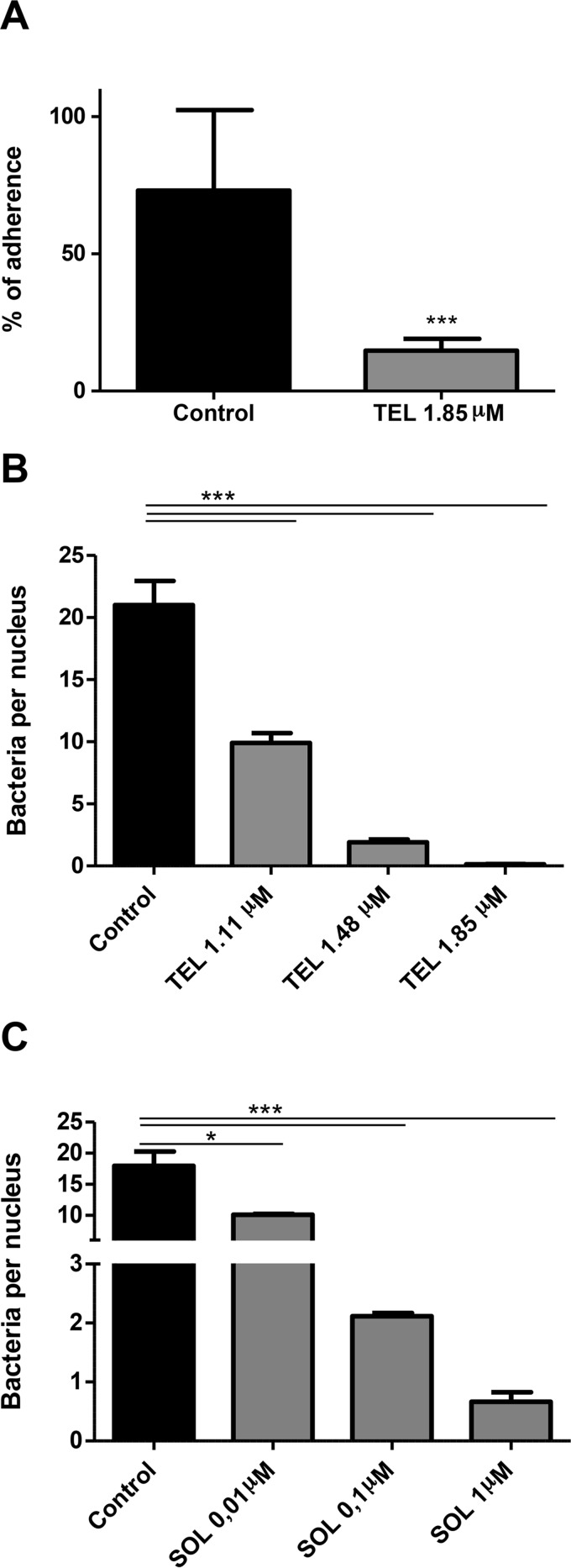
Inhibition of bacterial adherence to Caco-2 cells in the presence of ketolides. Semiconfluent monolayers of Caco-2 cells were infected with E. coli O157:H7 ZAP198 at an MOI of 20 in the presence or the absence of telithromycin (TEL) or solithromycin (SOL), and the percentage of adherent bacteria (A) or the number of bacteria per nucleus (B and C) was determined as detailed in Materials and Methods. (A) Adherent bacteria were determined by counting the number of CFU that adhered to Caco-2 cells after incubation with medium alone (control) or medium supplemented with 1.85 μM telithromycin. Results are expressed as the mean percentage relative to the amount of seeded bacteria ± SEM. ***, *P* < 0.001, Student's *t* test. (B and C) Caco-2 cells were infected with ZAP198 in the presence of the indicated concentration of telithromycin (B) or solithromycin (C). The mean number of adherent bacteria per nucleus ± SEM was determined by fluorescence microscopy. The results of a representative experiment out of two that were performed are shown. Four slides per treatment and at least 24 fields per slide were counted. *P* < 0.0001, ANOVA; ***, *P* < 0.001, Newman-Keuls multiple-comparison test; *, *P* < 0.05, Newman-Keuls multiple-comparison test.

Attachment to epithelial cells is a key stage of EHEC infection that allows colonization of the host intestinal tract, and T3SS is important, if not essential, for this process. Therefore, we wanted to examine whether the inhibition of T3SS determined by these ketolides had any effect on bacterial adherence to intestinal epithelial cells *in vitro*. We tested this hypothesis by culturing Caco-2 cells together with E. coli O157:H7 ZAP198 in the presence or the absence of 1.85 μM telithromycin. After 3 h of incubation, the percentage of attached bacteria was significantly reduced in the presence of telithromycin (Fig. 3A). This result was confirmed by quantifying the number of bacteria per nucleus by fluorescence microscopy. The number of bacteria per nucleus was reduced in a dose-dependent manner in the presence of telithromycin (Fig. 3B) or solithromycin (Fig. 3C).

**FIG 4:**
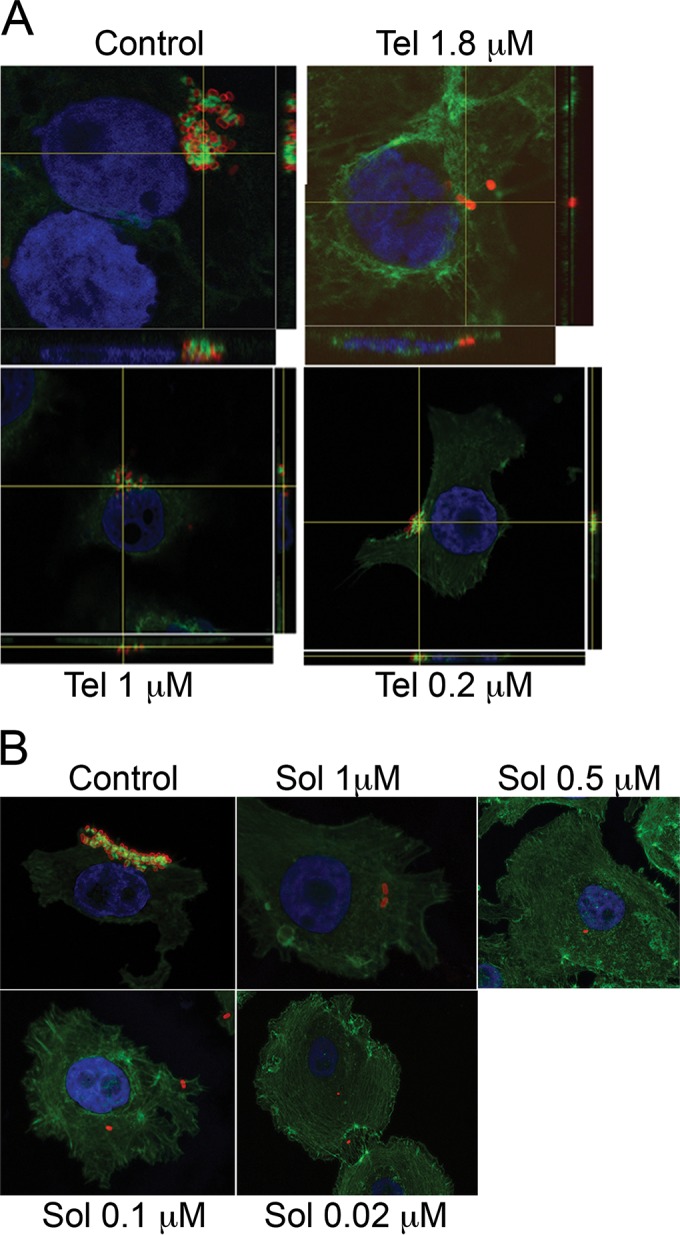
Inhibition of A/E lesion formation by ketolides. Semiconfluent monolayers of Caco-2 cells were infected with ZAP198 at an MOI of 20 in the presence or the absence of the indicated concentrations of telithromycin (Tel) or solithromycin (Sol). After 4 or 5 h of incubation, cells were fixed in 4% PFA. Bacteria were stained with anti-O157 antigen detected with PE-conjugated secondary antibody, nuclei were stained with DAPI, and actin polymerization was detected with FITC-conjugated phalloidin. Orthogonal sections show actin polymerization beneath adherent bacteria, which is typical of A/E lesions. Confocal images were acquired as described in Materials and Methods.

As a reduction in T3SS expression and in the number of adherent bacteria to Caco-2 cells was measured in the presence of the two ketolides, the impact on A/E lesion formation was examined by imaging actin cytoskeleton condensation under attached bacteria using FITC-phalloidin. Although some bacteria were still attached to cells in the presence of 1.85 μM telithromycin, there was no evidence of actin polymerization beneath these adherent bacteria, while this was clearly observed in controls to which no antibiotic had been added (Fig. 4). In addition, there were observable differences in the sizes of microcolonies when telithromycin was present in the culture medium; i.e., the attached microcolonies consisted of lower numbers of individual bacteria in the presence of the antibiotic than in its absence. At lower concentrations of telithromycin (1.00 and 0.02 μM), A/E lesions were observed (Fig. 4), although the extent of actin aggregation at 1.00 μM appeared to be less than that in the absence of the antibiotic, however, this was not quantified. On the other hand, solithromycin showed a wider range of inhibition of A/E lesion formation, since there were no A/E lesions observed at any of the concentrations tested (Fig. 4).

### Expression of the T3SS increases the susceptibility of E. coli O157 to solithromycin.

**FIG 5:**
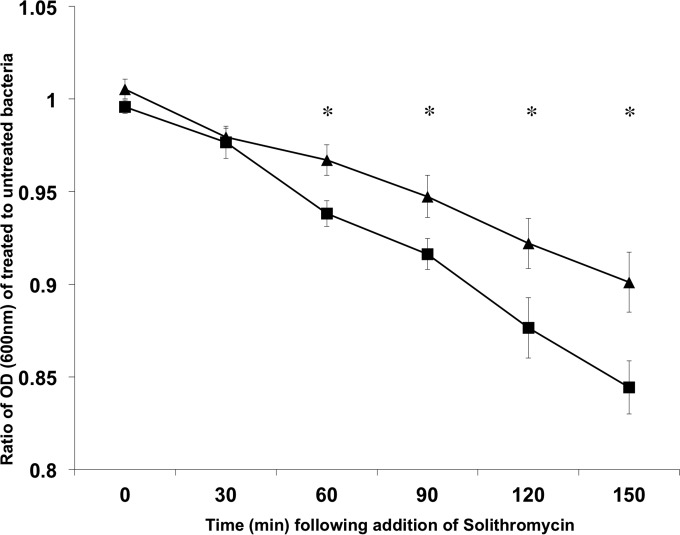
Type III secretion-dependent susceptibility to solithromycin. E. coli O157 strain ZAP193 (■) and its isogenic Δ*LEE2* mutant (▲) were cultured to an OD_600_ of ∼0.5 in MEM-HEPES to induce expression of the T3SS in the wild-type strain. The cultures were then split and 3 μM solithromycin was added to one of the culture pairs. Growth was then monitored at 30-min intervals, and the ratio of treated bacteria to untreated bacteria was plotted for 6 pairs of cultures for each strain. The results are expressed as the mean ± SEM. *, *P* < 0.05, two-sample *t* test.

To determine if expression of the T3SS impacts the survival of the bacteria when exposed to the ketolide, E. coli O157 (ZAP193) and its mutant isogenic with an *LEE2* operon deletion (Δ*LEE2* mutant) were cultured in MEM-HEPES to an OD_600_ of ∼0.5 in MEM-HEPES, in order to induce the T3SS, if it was intact. At that point, cultures were split in two and 3 μM solithromycin was added to only one of the pair. It was evident that the wild type was inhibited to a greater extent than the mutant by addition of the antibiotic, and this was most evident at the later time points (Fig. 5). A similar and significant effect on the overnight growth of strain ZAP198 and its isogenic *ler* mutant was seen following addition of 1.5 μM solithromycin (*P* < 0.05; data not shown).

### Preexposure of epithelial cells to solithromycin reduces T3SS-dependent attachment of E. coli O157.

**FIG 6:**
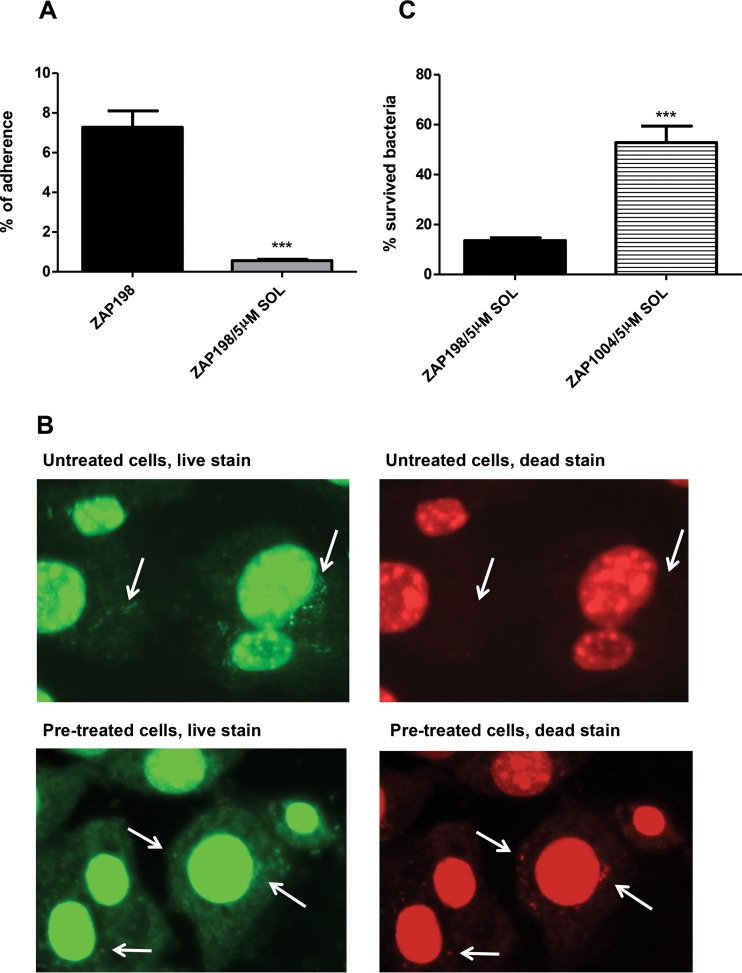
Preincubation of bovine epithelial (EBL) cells with solithromycin reduces bacterial recovery in a T3S-dependent manner. (A) The percentage of ZAP198 bacteria recovered from EBL cells with and without a 3-h pretreatment with 5 μM solithromycin (SOL). The results represent the mean data from three separate experiments with technical repeats. ***, *P* < 0.0001, Student's *t* test. (B) Live/dead staining of ZAP198 added to EBL cells either pretreated or not with 5 μM solithromycin for 3 h. Bacteria were added at a reduced MOI of 20:1 compared with the MOI used in the assays providing the binding data (100:1). All bacteria should stain green, whereas bacteria that have lost cell wall/membrane integrity also stain red. Arrows, examples of individual or grouped bacteria. Dead bacteria were imaged only with the cells pretreated with solithromycin. (C) The percentage of ZAP198 (wild type) and ZAP1004 (Δ*ler* derivative) bacteria recovered from EBL cells pretreated for 3 h with 5 μM solithromycin as described in Materials and Methods. ***, *P* < 0.0005, Student's *t* test.

One property of ketolides is that they are actively taken up into eukaryotic cells and so can accumulate to relatively high levels in the cytoplasm (36, 37). We therefore tested whether preexposure of epithelial cells to solithromycin could alter the capacity of E. coli O157 to attach to the cells and whether opening of translocation channels into these preloaded cells may result in a conduit for the antibiotic and bacterial killing. The epithelial cells were washed after antibiotic exposure to limit the amount of freely available ketolide. Cells initially exposed to 5 μM solithromycin had bacterial binding levels reduced by at least 10-fold (Fig. 6A). Furthermore, the use of live/dead staining indicated that a proportion of the attached bacteria were being killed when binding to the preexposed epithelial cells (Fig. 6B). The reduced level of bacterial binding likely reflects both reduced attachment and higher killing. To investigate if the findings were simply a result of the general release of ketolide from the epithelial cells, we determined how the recovery of bacteria with and without a T3SS would be affected by epithelial cells preexposed to solithromycin. The experiments were carried out as coinfections with E. coli O157 ZAP198 (Nal^r^) and ZAP198 Δ*ler* (Amp^r^), which were independently enumerated. While bacteria with the *ler* deletion did not bind to the epithelial cells as well, they could be recovered, and the ratios of the two strains were determined with and without preexposure of the cells to solithromycin. It was evident that the wild-type strain expressing a T3SS was much more susceptible to the preexposed cells, indicating that the killing was again T3S dependent (Fig. 6C).

### Effect of ketolides on SOS induction.

**FIG 7:**
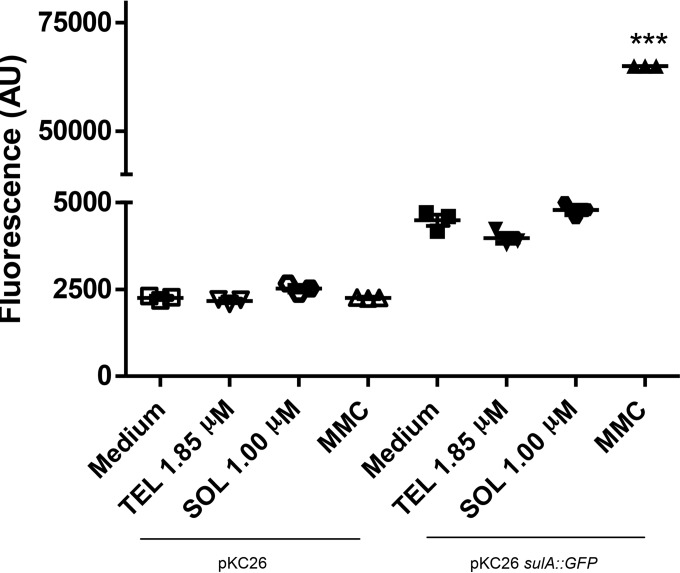
Ketolides do not induce DNA damage. The *sulA* promoter was fused with the gene for GFP and transformed into ZAP198 (pKC26 *sulA*::*gfp*). The fluorescence and OD_600_ were determined after 6 h of culture. ZAP198 transformed with a promoter-less GFP plasmid (pKC26) was used as a background control under each culture condition. Mitomycin C (MMC) was used as positive control since it induces DNA damage and, consequently, SulA expression. Lines represent the averages of three biological repeats. The results of one representative experiment out of at least two that were performed are shown. OD_600_, optical density at 600 nm; AU, arbitrary units. ***, *P* < 0.0001, ANOVA.

Antibiotics are generally not recommended as a treatment for EHEC-associated gastrointestinal infections since certain classes can induce DNA damage and activate the bacterial SOS system, which can promote Stx production. In order to assess whether telithromycin or solithromycin induced the SOS response, a GFP reporter fusion to the *sulA* promoter was used. SulA expression is induced as a response to DNA damage (38, 39), so *sulA* induction is an indirect measure of the SOS response. As a negative control, vector pKC26, which contains *gfp* but no cloned promoter, was used. As a positive control, pKC26 carrying the SulA reporter was used in a strain treated with 1 μg/ml mitomycin C (MMC), since MMC induces interstrand DNA cross-links (40), causing double-strand breaks, which induce the SOS response (41). No increase in the level of the SulA reporter was measured with treatment with either telithromycin or solithromycin at concentrations shown to limit T3S, indicating no induction of the SOS response (Fig. 7).

## DISCUSSION

Enterohemorrhagic Escherichia coli (EHEC) infection is a worldwide human disease with life-threatening systemic complications, such as hemolytic-uremic syndrome (HUS) (42, 43) and neurological damage (44, 45) related to Shiga toxin (Stx) production. EHEC strains were originally defined to be strains that produce Stx and express a T3SS, as well as to be isolated from patients exhibiting specific symptoms, such as hemorrhagic diarrhea. At present, there is no preventative treatment that can lessen the risk of serious clinical complications, such as HUS. Management and treatment rely purely on supportive therapy (46–49). In the present study, the ketolide telithromycin and the related fluoroketolide solithromycin were demonstrated to repress T3SS at concentrations of the antibiotics at which the growth of the bacteria still occurred, although at slightly reduced rates. This indicates that at low antibiotic concentrations, translation of the T3SS might be preferentially inhibited relative to that of other bacterial proteins in the cell. This is supported by analysis of translational fusions to the different operons encoded on the LEE (50). Reduced expression of LEE2, LEE4, and LEE5 was observed, while the expression of LEE1 or RpsM (a ribosomal control protein) was relatively unaffected. The differential repression of the different reporters was an unexpected finding, and the basis for the variation in translational control between the operons is currently unknown, although we have observed coupled posttranscriptional regulation of the *LEE4* and *LEE5* operons but not the *LEE1* operon (33, 34). We speculate that the assembly of the T3SS is a staged process that may require localized coupling of translation to assembly and secretion. It is possible that *LEE1* may not be under such control, whereas later operons are. This could account for the differential activity of the translational reporters used in this study (Fig. 2).

The T3SS has been recognized to be a key virulence factor for disease progression (51), as intestinal colonization is likely to be important for EHEC infection. The T3SS is required for the formation of characteristic A/E lesions, identified by localized effacement of microvilli and intimate adherence of bacteria to the apical plasma membrane, often with the formation of actin-rich pedestal-like structures beneath the bacteria, a process that has been linked to disease (52). The two ketolides tested in the current study led to reduced levels of bacterial adherence to Caco-2 cells and a diminished capacity to form A/E lesions. These results are in agreement with the reduced secretion of T3SS proteins and inhibition of LEE2, LEE4, and LEE5 translational reporters by treatment with sub-MIC levels of both antibiotics. These findings indicate that ketolide use should result in a diminished pathogenicity of EHEC *in vivo*, although separation of this from an impact on bacterial growth makes it difficult to justify animal studies. Our data are in agreement with data from previous research demonstrating the therapeutic benefits of macrolide treatment on EHEC infections. Two independent studies have shown that azithromycin (53) and rokitamycin (54) treatment reduces lethality and Stx-related systemic alterations in different animal models of EHEC infection. However, these studies link the clinical benefit of this therapy only to a reduction in the levels of Stx production. Our findings suggest that macrolides/ketolides could also contribute to reductions in the levels of EHEC carriage and possibly also reductions in the rates of HUS development by reducing T3SS protein production. In this sense, previous studies have demonstrated that the degree of gut adhesion correlates with the ability to cause disease (55, 56). In cattle, the main reservoir host for EHEC O157, deletion of the *LEE4* operon prevents gastrointestinal colonization (57).

Our results also demonstrate another advantage of using the ketolides, in that these antibiotics are known to accumulate in eukaryotic cells (36, 37). This raises the possibility that when the bacteria open up a channel via the T3SS into a cell pretreated with the antibiotic, they are potentially exposed to the compound. Cells pretreated with solithromycin and then washed were able to significantly reduce E. coli O157 adherence compared to that obtained on untreated cells, and this was at least in part due to killing of the bacteria on exposure to the pretreated cells. This lower recovery of bacteria was T3SS dependent, as the effect was significantly diminished for a *ler* mutant. There was also evidence that even in the absence of eukaryotic cells but in a medium that promotes T3SS expression, bacteria capable of expressing the system were more susceptible. This effect was not as dramatic as that observed on the preloaded eukaryotic cells, but this may represent the different exposure concentrations and the status of the export channel, which may be more restricted for antibiotic entry in the absence of target cells. One report previously demonstrated the T3SS dependence on antibiotic activity for Shigella (58), and the authors of that report proposed that the antibiotics may be gaining entry via the export system, although they provided no evidence for this. However, in their work, pretreatment of cells did not impact Shigella invasion. We can propose from the current study that while antibiotics like the ketolides are considered effective for some intracellular bacteria, bacteria that open up a channel to the cell, such as EPEC and EHEC, generally without invasion, can also potentially be targeted by accumulated intracellular antibiotic.

The use of antimicrobial agents in EHEC infections has been controversial, since certain classes can induce the bacteriophage(s) that encodes Stx, thus leading to Stx production. In particular, it has been demonstrated that antibiotics which interfere with DNA replication, such as the quinolone antibiotic ciprofloxacin and trimethoprim-sulfamethoxazole, induce Stx production *in vitro* and in animal models *in vivo* (26, 59–61). This fact supports the epidemiological observation that treatment of EHEC infections with these antibiotics increases the risk of development of HUS (62–64). Stx is encoded by genes within the late-gene region of temperate bacteriophages integrated in the bacterial chromosome, which can be induced by DNA-damaging agents, such as UV light or antibiotic exposure, thus prompting an SOS response, whereby the production of Stx increases (24–27). In the presence of DNA-damaging agents, an activated RecA acts as a coprotease in the cleavage of the LexA repressor, allowing the transcription of the SOS genes (65, 66). The transcription of *sulA*, which is one of the SOS genes, has been used as a bioreporter for the microbial cytotoxicity of different DNA-damaging agents (39, 67). Our results show that telithromycin and solithromycin do not induce *sulA* expression when used at sub-MICs, thus suggesting that Stx production is not induced with these antibiotics. This finding is in agreement with the findings of previous studies showing that protein synthesis inhibitors suppress Stx release from EHEC (53, 61, 68–71). Alternatively, about 10% of human intestinal isolates are susceptible to Stx-encoding phages that could contribute to Stx production (72, 73), and ketolides would also interfere with that transfer (68).

In conclusion, our study suggests that certain ketolides and their derivatives warrant further testing as possible therapeutic options for dealing with primary exposure cases in EHEC outbreaks and may have wider value for targeting other bacterial pathogens with T3SSs.
